# Supplementary material for: Integrable quantum many-body sensors for AC field sensing
Source: Sci Rep. 2022 Aug 30;12:14760. doi: 10.1038/s41598-022-17381-y (PMC9427993; doi:10.1038/s41598-022-17381-y)
Supplement: Supplementary file 1 — Supplementary Information. [file 41598_2022_17381_MOESM1_ESM.pdf]

Supplementary Material for Integrable Quantum Many-Body Sensors for AC Field Sensing

Utkarsh Mishra[1] and Abolfazl Bayat[1]
[1]*Institute of Fundamental and Frontier Sciences, University of Electronic Science and Technology of China, Chengdu 610054, China*

FLOQUET HAMILTONIAN

We present here the analytical description of the model

$$H(t) = -J\sum_{i=1}^{N}\hat{\sigma}_i^x\hat{\sigma}_{i+1}^x - \sum_i (h_0 + h(t))\hat{\sigma}_i^z, \tag{S1}$$

where, $J > 0$ is the nearest-neighbor spin-spin interaction, h_0 is a DC external magnetic field which is tunable, $\hat{\sigma}_i^{x/y}$ are Pauli matrices at site i, and the periodic boundary conditions is assumed, i.e., $\hat{\sigma}_{N+1}^{x/y} = \hat{\sigma}_1^{x/y}$. The system represents interacting spin-1/2 particles with Ising type interaction on a $1D$ lattice of length N in a transverse field to serve as a many-body probe for sensing a time-periodic magnetic field, $h(t)$. The Hamiltonian in Eq. (S1) can be solved analytically [S1, S2]. The first step is to map the spin operators, $\hat{\sigma}_i$, into fermionic operators, $\hat{c}_i^\dagger(\hat{c}_i)$, defined via the following transformations:

$$\begin{aligned}\hat{\sigma}_j^- &= e^{i\pi\sum_{i=1}^{j-1}\hat{\sigma}_i^+\hat{\sigma}_i^-}\hat{c}_j \\ \hat{\sigma}_j^+ &= \hat{c}_j^\dagger e^{-i\pi\sum_{i=1}^{j-1}\hat{\sigma}_i^+\hat{\sigma}_i^-},\end{aligned} \tag{S2}$$

where, $\hat{\sigma}_j^\pm = (\hat{\sigma}_j^x \pm \hat{\sigma}_j^y)/2$. The Hamiltonian as a result transformed into a quadratic-fermionic form

$$\begin{aligned}H(t) &= -J\sum_{i=1}^{N}(\hat{c}_j^\dagger\hat{c}_{j+1}^\dagger + \hat{c}_{j+1}\hat{c}_j + \hat{c}_j^\dagger\hat{c}_{j+1} + \hat{c}_{j+1}^\dagger\hat{c}_j) \\ &- h(t)\sum_i(2\hat{c}_j^\dagger\hat{c}_i - 1).\end{aligned} \tag{S3}$$

It is to be noted that the fermionic Hamiltonian in Eq. (S3) is transnational invariant and therefore by applying a Fourier transformation $\hat{c}_j = \frac{1}{\sqrt{N}}\sum_k e^{ikj}\hat{c}_k$, it can be written in $k-$space. The full Hamiltonian can be decomposed into the sum of the Hamiltonian for each $k-$mode i.e., $H = \sum_k H_k$ with H_k being the Hamiltonian of the kth subspace given by $H_k = \sum_{k>0}(h(t) + J\cos(k))\left(\hat{c}_k^\dagger\hat{c}_k - \hat{c}_{-k}\hat{c}_{-k}^\dagger\right) + J\sin(k)(\hat{c}^\dagger\hat{c}_{-k}^\dagger - \hat{c}_k\hat{c}_{-k})$. It can be seen that each H_k acts on a subspace spanned by basis $\{|0\rangle, c_k^\dagger c_{-k}^\dagger|0\rangle\}$, where $|0\rangle$ is the vacuum of the Jordan-Wigners fermions $\hat{c}_k$. By defining pseudo spin basis as $|\uparrow\rangle_k = |0\rangle$ and $|\downarrow\rangle_k = c_k^\dagger c_{-k}^\dagger|0\rangle$, we can write H_k as

$$H_k = (J\cos(k) + h(t))\,\hat{\sigma}_p^z + J\sin(k)\hat{\sigma}_p^y, \tag{S4}$$

where $\hat{\sigma}_p^y$ and $\hat{\sigma}_p^z$ are pseudospin operators in the pseudospin basis and k is termed as quasi-momentum which takes the values $k = \frac{\pi}{N}, \frac{3\pi}{N}, \ldots, \frac{(N-1)\pi}{N}$ for even N. The Hamiltonian in Eq. (S1) can be decomposed into the sum of even and odd parity-conserving Hamiltonians and the ground state of the system belongs to the even parity subspace for every finite N and it assumes BCS like form, given by [S3]

$$|\Psi(0)\rangle = \prod_{k>0}(u_k(0) + v_k(0)c_k^\dagger c_{-k}^\dagger)|0\rangle, \tag{S5}$$

where $u_k = \sin(\theta/2)$, $v_k = \cos(\theta/2)$, and $\theta = \tan^{-1}\frac{J\sin(k)}{h_0 + J\cos(k)}$. Thus, when $u_k = 1, v_k = 0$ for all k, it corresponds to a state with all spins in the eigenbasis of $\hat{\sigma}^z$ with eigenvalue +1. Under the dynamics given by the Hamiltonian in Eq. (S3) the states in the two parity sectors as well as those with different momentum evolves independently. Thus, for the unitary time dynamics, it is enough to consider states $\{|0\rangle, c_k^\dagger c_{-k}^\dagger|0\rangle\}$ which leads to time evolved state in the form [S4]

$$|\Psi(t)\rangle = \prod_{k>0}(u_k(t) + v_k(t)c_k^\dagger c_{-k}^\dagger)|0\rangle, \tag{S6}$$

where $u_k(t)$ and $v_k(t)$ are the solutions of the Schrödinger equation

$$i\hbar\frac{d}{dt}(u_k, v_k)^T = H_k(t)(u_k, v_k)^T. \tag{S7}$$

The above analysis applies to any general time-dependent function $h(t)$. For stroboscopic dynamics, i.e., time-evolution of the system monitored in the steps $t = n\tau$, the state of the system at any time $t = n\tau$, can be obtained by repeated application of the unitary operator as

$$|\Psi(t)\rangle = [U(\tau)]^n|\Psi(0)\rangle, \tag{S8}$$

where $U(\tau) = \mathcal{T}e^{-i\int_0^\tau H(t)dt}$, is the the time-evolution operator for single time-period τ and $\mathcal{T}$ denotes time ordered product. For the Dirac-delta function, it is possible to find the effective Hamiltonian, known as Floquet Hamiltonian H^F, which generates equivalent dynamics. Therefore, the equation for the ensuing dynamics, namely Eq. (S8), can be simplified in term of the Floquet Hamiltonian as

$$\begin{aligned}|\Psi(t = n\tau)\rangle &= e^{-inH^F\tau}|\Psi(0)\rangle \\ &= \prod_{k>0} e^{-inH_k^F\tau}|\psi_k(0)\rangle,\end{aligned} \tag{S9}$$

where $|\psi_k(0)\rangle = (u_k(0) + v_k(0)c_k^\dagger c_{-k}^\dagger))|0\rangle$. Once the time-dependent state $|\Psi(t = n\tau)\rangle$ is known, the time-dependent magnetization at the stroboscopic time, $t = n\tau$, is expressed as $m_z(n\tau) = \langle\Psi(n\tau)|\frac{1}{N}\sum_{i=1}^N \hat{\sigma}_i^z|\Psi(n\tau)\rangle$. By employing the Jordan-Wigner transformation and expressing $\hat{\sigma}^z$ in terms of $\hat{\sigma}^\pm$, we have

$$m_z(n\tau) = \frac{1}{N}\sum_{k>0}\Big(|u_k(n\tau)|^2 - |v_k(n\tau|^2), \tag{S10}$$

where, $u_k(n\tau)$ and $u_k(n\tau)$ are solutions of the Schrödinger equation given in Eq. (S7).

QUANTUM FISHER INFORMATION OF A BLOCK OF SIZE L

To calculate the quantum Fisher information of a subsystem of size L, we need to calculate the reduced density matrix of L sites (we consider L contiguous sites). The calculation of reduced density matrix requires partial tracing of complimentary degrees of freedoms. For quadratic Hamiltonians of the form given in Eq. (S3), this is accomplished by noting the relation between the density matrix and the matrix of single-particle correlations of L sites [S5]. The form of the reduced density matrix which reproduces correct correlation matrix on L sites is given by $\rho_L = e^{-\Omega}/Z$, where Ω is quadratic in fermionic operators with energies ϵ_i i.e., $\Omega = \sum_{i=1}^L \epsilon_i\hat{c}_i^\dagger\hat{c}_i$ and Z is the normalization constant. By breaking the complex fermions into Majorana basis defined as $\hat{c}_i = \frac{1}{2}(a_{2i-1} + ia_{2i})$ and $\hat{c}_i^\dagger = \frac{1}{2}(a_{2i-1} - ia_{2i})$, the density matrix of blocks of size L can be represented as free fermionic Gaussian state, given by

$$\rho_L = \frac{e^{-\frac{i}{4}\vec{a}^T\Omega\vec{a}}}{Z}, \tag{S11}$$

where, $Z = \mathrm{Tr}[e^{-\frac{i}{4}\vec{a}^T\Omega\vec{a}}]$. Here Ω is a $2L\times 2L$ real antisymmetric matrix and $\vec{a} \equiv (a_1, \ldots, a_{2L})^T$ is a $2L$- dimensional array of Majorana fermions. The Gaussian state in Eq. (S11) is completely characterized by a two-point correlation matrix Γ whose elements are given by

$$\Gamma_{ij} = \mathrm{Tr}(\rho_L a_i a_j). \tag{S12}$$

The Γ matrix is an antisymmetric matrix. The elements of the Γ matrix can be obtained in terms of $\mathcal{C}$ and $\mathcal{I}$ matrix where, $C_{i,j} = \mathrm{Tr}[\rho_L\hat{c}_i^\dagger\hat{c}_j]$ and $\mathcal{I}_{i,j} = \mathrm{Tr}[\rho_L\hat{c}_i^\dagger\hat{c}_j^\dagger]$. In terms of these matrices, we have [S6]

$$\begin{aligned}\Gamma_{2i-1,2j-1} &= \delta_{i,j} + 2i\Im[C_{i,j} + \mathcal{I}_{i,j}] \\ \Gamma_{2i-1,2j} &= i\delta_{i,j} - 2i\Re[C_{i,j} - \mathcal{I}_{i,j}] \\ \Gamma_{2i,2j-1} &= -i\delta_{i,j} + 2i\Re[C_{i,j} + \mathcal{I}_{i,j}] \\ \Gamma_{2i,2j} &= \delta_{i,j} + 2i\Im[C_{i,j} - \mathcal{I}_{i,j}],\end{aligned}$$

where, $\Re[\cdot]$ ($\Im[\cdot]$) represents the real (imaginary) part, $i, j = 1, \ldots, L$, $\delta_{i,j}$ is discrete Kronecker delta function, and the elements of the time-dependent correlation matrix, $C(t)$, and anomalous correlation matrix, $\mathcal{F}(t)$, are given as

$$\begin{aligned} C_{i,j} &= \langle \Psi(t)|\hat{c}_i^\dagger \hat{c}_j|\Psi(t)\rangle \\ &= \frac{2}{N}\sum_k |u_k(t)|^2 \cos(k(j-i)), \end{aligned} \tag{S13}$$

and

$$\begin{aligned} \mathcal{I}_{i,j} &= \langle \Psi(t)|\hat{c}_i^\dagger \hat{c}_j^\dagger|\Psi(t)\rangle \\ &= \frac{2i}{N}\sum_{k>0} u_k^*(t) v_k(t) \sin(k(j-i)). \end{aligned} \tag{S14}$$

Once the Γ matrix is known, the quantities of interest can be expressed in terms of the Γ matrix. For example, the symmetric logarithmic derivative has been obtained for the Gaussian states, Eq. (S11), and in the Majorana basis it has the following form [S7]

$$\hat{L} = \frac{1}{2}\vec{a}^T K \vec{a} + \vec{\zeta}^T \vec{a} + \Lambda, \tag{S15}$$

where K is a Hermitian anti-symmetric matrix of dimension $2L \times 2L$, ζ is a real vector and Λ is a real number. The matrix elements of the K in the eigenbasis of $\Gamma = \sum_{r=1}^{2L} \gamma_r |\gamma_r\rangle\langle\gamma_r|$ are given by [S7]

$$K_{rs} = \frac{(\partial_{h_1}\Gamma)_{rs}}{(\gamma_r\gamma_s - 1)}, \tag{S16}$$

with $(\partial_{h_1}\Gamma)_{rs} = \langle r|\partial_{h_1}\Gamma|s\rangle$ and the partial derivative is taken with respect to the parameter to be estimated. By substituting $\hat{L}$ into $\mathrm{Tr}[\rho_L \hat{L}^2]$, the QFI in the eigenbasis of Γ is expressed as [S7, S8]

$$F_Q = \sum_{r,s=1}^{2L} \frac{\langle r|\partial_{h_1}\Gamma|s\rangle\langle s|\partial_{h_1}\Gamma|r\rangle}{(1-\gamma_r\gamma_s)}. \tag{S17}$$

We have used this expression to obtain the Fisher information of a block of size L in our time-dependent model. The expression of QFI is valid for all parameters except when $\gamma_r = \gamma_s = \pm 1$. At these values of $\gamma' s$, the density matrix ρ_L becomes singular and is not well defined.

OPTIMAL VERSUS SUB-OPTIMAL MEASUREMENTS

In order to obtain the exact form of the optimal POVM, we first define the symmetric logarithmic derivative operator $\hat{L}$ to satisfy

$$\partial_{h_1}\rho_L = \frac{\hat{L}\rho_L + \rho_L\hat{L}}{2}. \tag{S18}$$

By inserting the spectral decomposition form of ρ_L in Eq. (S18), one can easily obtain the following expression for $\hat{L}$

$$\begin{aligned} \hat{L} &= \sum_p \frac{\partial_{h_1}\lambda_p}{\lambda_p}|\lambda_p\rangle\langle\lambda_p| \\ &+ \sum_{p\neq q} \frac{\lambda_p - \lambda_q}{\lambda_p + \lambda_q}\langle\lambda_p|\partial_{h_1}\lambda_q\rangle|\lambda_p\rangle\langle\lambda_q|, \end{aligned}$$

where, $|\partial_{h_1}\lambda_p\rangle = \frac{\partial|\lambda_p\rangle}{\partial h_1}$. The QFI then can be written as $F_Q(h_1) = \mathrm{Tr}[\rho_L \hat{L}^2]$ and the eigenvectors of $\hat{L}$ provide the optimal POVM projectors $\{\Pi_r\}$ [S9]. In general, the symmetric logarithmic derivative operator $\hat{L}$ and thus its

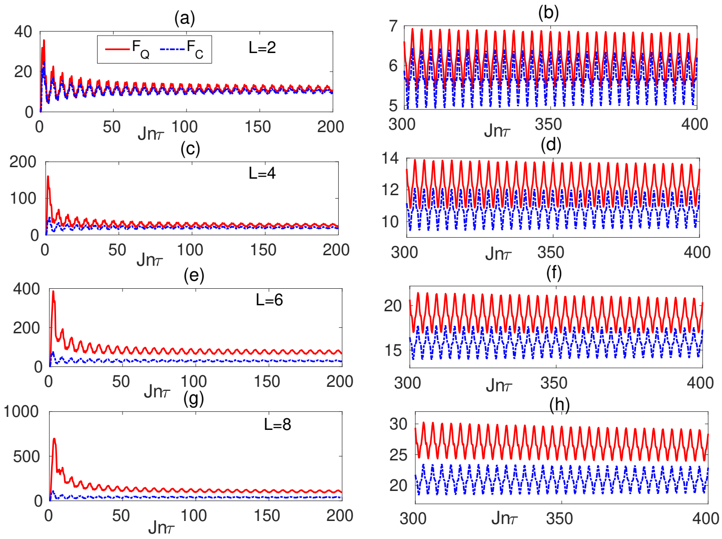

FIG. S1: The QFI of a block of size L=2, 4, 6, 8 is compared with the CFI resulted from the block magnetization measurement when the DC field is tuned at h_0/J=1.0. The plots are given for two different values of h_1, namely h_1/J=0.1 (the left panels) and h_1/J=0.2 (the right panels). Here $J\tau = 0.2$ and $N = 2000$.

eigenvectors, which are the optimal measurement basis, depend on the unknown parameter h_1. Therefore, finding the optimal measurement basis is one of the big challenges in quantum estimation theory which often hinders saturating the quantum Cramér-Rao bound. In fact, in most of the cases, the bound is only achievable when sophisticated adaptive methods for updating the measurement basis are employed [S10–S14].

Here we outline the calculation of symmetric logarithmic derivative for a single and two-qubit density matrix. A general single-qubit state of the system is written as $\rho_1 = \frac{1}{2}(\mathbb{I} + \vec{m}.\vec{\sigma})$, where $\vec{m} = \mathrm{Tr}(\rho_1\vec{\sigma})$ and $\vec{\sigma} = (\hat{\sigma}^x, \hat{\sigma}^y, \hat{\sigma}^z)$. It is to be noted that the Hamiltonian $H(t)$ is invariant under fermionic parity transformation, i.e., $H(t) = \Big(\prod_{j=1}^{L}\hat{\sigma}^z\Big)H(t)\Big(\prod_{j=1}^{L}\hat{\sigma}^z\Big)$. This implies, as shown in [S15, S16], that $m^x, m^y = 0$. Thus, we get a single-site density matrix which is diagonal in the eigenbasis of $\hat{\sigma}^z$ i.e., in $\{|\uparrow\rangle, |\downarrow\rangle\}$ basis. The symmetric logarithmic derivative in this basis is given by $\hat{L} = \frac{(\partial_{h_1}p)^2}{p}|\uparrow\rangle\langle\uparrow| + \frac{(\partial_{h_1}(1-p))^2}{1-p}|\downarrow\rangle\langle\downarrow|$, where $p = (1+m_z)/2$. The quantum Fisher information can be calculated using $F_Q = \mathrm{Tr}[\rho_1\hat{L}^2]$ and it turns out as

$$F_Q = \frac{(\partial_{h_1}m_z)^2}{(1+m_z)(1-m_z)}. \tag{S19}$$

On the other hand, the eigenvectors of $\hat{L}$ are $\{|\uparrow\rangle, |\downarrow\rangle\}$. If the set of POVM is constructed using the projections onto the eigenvectors of $\hat{L}$, then the classical Fisher information F_C is given by

$$F_C = \frac{1}{p_\uparrow}(\langle\uparrow|\partial_{h_1}\rho_1|\uparrow\rangle)^2 + \frac{1}{p_\downarrow}(\langle\downarrow|\partial_{h_1}\rho_1|\downarrow\rangle)^2, \tag{S20}$$

where, $p_\uparrow = \langle\uparrow|\rho_1|\uparrow\rangle$ and $p_\downarrow = \langle\downarrow|\rho_1|\downarrow\rangle$. A further simplification of F_C gives $F_C = F_Q$.

For the density matrix of two nearest-neighbor sites, one also needs to calculate the two-point correlators $\hat{\sigma}_i^s \otimes \hat{\sigma}_{i+1}^{s'}(s, s' = x, y, z)$. It can be seen that the correlators such as $\hat{\sigma}_i^x \otimes \hat{\sigma}_{i+1}^z$ and $\hat{\sigma}_i^y \otimes \hat{\sigma}_{i+1}^z$ vanishes due to invariance of the Hamiltonian under parity transformation [S17, S18]. Since periodic boundary conditions are assumed, the nearest-neighbor state is independent of which two neighboring sites are chosen for constructing the density matrix. The two-site density matrix of the system, therefore, is given by

$$\rho_2(t) = \begin{pmatrix} u_{11} & 0 & 0 & u_{14} \\ 0 & u_{22} & u_{23} & 0 \\ 0 & u_{32} & u_{33} & 0 \\ u_{41} & 0 & 0 & u_{44} \end{pmatrix} \tag{S21}$$

where,

$$
\begin{aligned}
u_{11} &= \frac{1}{4}\left[1+2\langle\hat{\sigma}^z\rangle+\langle\hat{\sigma}_i^z\hat{\sigma}_{i+1}^z\rangle\right] \\
u_{22} &= u_{33}=\frac{1}{4}\left[1-\langle\hat{\sigma}_i^z\hat{\sigma}_{i+1}^z\rangle\right] \\
u_{23} &= \frac{1}{4}\left[\langle\hat{\sigma}_i^x\hat{\sigma}_{i+1}^x\rangle+\langle\hat{\sigma}_i^x\hat{\sigma}_{i+1}^y\rangle+\langle\hat{\sigma}_i^y\hat{\sigma}_{i+1}^x\rangle+\langle\hat{\sigma}_i^y\hat{\sigma}_{i+1}^y\rangle\right] \\
u_{14} &= \frac{1}{4}\left[\langle\hat{\sigma}_i^x\hat{\sigma}_{i+1}^x\rangle-\langle\hat{\sigma}_i^x\hat{\sigma}_{i+1}^y\rangle-\langle\hat{\sigma}_i^y\hat{\sigma}_{i+1}^x\rangle-\langle\hat{\sigma}_i^y\hat{\sigma}_{i+1}^y\rangle\right] \\
u_{44} &= \frac{1}{4}\left[1-2\langle\hat{\sigma}^z\rangle+\langle\hat{\sigma}_i^z\hat{\sigma}_{i+1}^z\rangle\right].
\end{aligned}
\tag{S22}
$$

The other non-zero elements are given as $u_{32}=u_{23}^*$, and $u_{41}=u_{14}^*$. The non-zero correlators $\hat{\sigma}_i^s\otimes\hat{\sigma}_{i+1}^{s'}$ can be obtained using the formalism presented in [S19]. Once the two-site density matrix is obtained, the symmetric logarithmic derivative for the two-qubit state can be calculated. We find that the symmetric logarithmic derivative with respect to the state $\rho_2(t)$ is given by

$$
\hat{L}(t)=\begin{pmatrix} L_{11} & 0 & 0 & L_{14} \\ 0 & 1 & -1 & 0 \\ 0 & 1 & 1 & 0 \\ L_{41} & 0 & 0 & L_{44}\end{pmatrix}. \tag{S23}
$$

The eigenvectors of symmetric logarithmic derivative are given by

$$
\begin{aligned}
|\ell_1\rangle &= c_1(h_0,h_1)|\uparrow\uparrow\rangle+c_2(h_0,h_1)|\downarrow\downarrow\rangle, \\
|\ell_2\rangle &= c_2(h_0,h_1)|\uparrow\uparrow\rangle+c_3(h_0,h_1)|\downarrow\downarrow\rangle, \\
|\ell_3\rangle &= \left(|\uparrow\downarrow\rangle-|\downarrow\uparrow\rangle\right)/\sqrt{2}, \\
|\ell_4\rangle &= \left(|\uparrow\downarrow\rangle+|\downarrow\uparrow\rangle\right)/\sqrt{2},
\end{aligned}
\tag{S24}
$$

where $c_1=-\frac{-L_{11}+L_{44}+\sqrt{(L_{11}-L_{44})^2+4L_{14}L_{41}}}{2L_{14}L_{41}}$ and $c_2=-\frac{-L_{11}+L_{44}-\sqrt{(L_{11}-L_{44})^2+4L_{14}L_{41}}}{2L_{14}L_{41}}$. As two of the eigenvectors namley $|\ell_1\rangle$ and $|\ell_2\rangle$ depends on the parameter to be estimated i.e., on h_1, the classical Fisher information obtained from the measurement of Eq. (S24) may not be equal to the quantum Fisher information [S20].

The QFI is only a bound in the Cramér-Rao inequality and it is not guaranteed to be saturated unless an optimal measurement basis, as well as an optimal estimator, are chosen. As mentioned before, the measurement basis is determined by eigenvectors of the symmetric logarithmic derivative operator $\hat{L}$. In a general case, the optimal measurement basis depends on the sensing parameter h_1 which by definition is unknown making the optimal sensing impractical. Normally, to overcome this problem, one has to update the measurement basis adaptively by extracting partial information about the unknown parameter using a sequence of non-optimal measurement basis [S10–S14]. Due to practical constraints, even if the optimal measurement basis is known (e.g. from an adaptive strategy) its implementation may not be feasible. Therefore, one of the desired issues in quantum metrology problems is to find a suitable measurement basis that is close to the optimal one and is independent of h_1.

We consider a simple, though sub-optimal, measurement which is independent of h_1. The measurement is the block magnetization, which for a block of size L takes $L+1$ outcomes from $O_1=+L$ (when all the qubits are $|\uparrow\rangle$), $O_2=L-2$ (when except one qubit the rest are in the state $|\uparrow\rangle$) until $O_{L+1}=-L$ (when all the qubits are $|\downarrow\rangle$). Each of the outcomes O_r appears with the probability p_r. Then one can get the corresponding classical Fisher information F_C. Note that the block magnetization is easily measurable in ion traps [S21–S23] and superconducting quantum devices [S24–S26]. In Figs. S1(a)-(h) we plot both the CFI, computed for the block magnetization, and the QFI as a function of time $t=n\tau$ for various block sizes. In all these plots the control field is fixed to be $h_0/J=1$ while the left and the right panels represent the results for $h_1/J=0.1$ and $h_1/J=0.2$, respectively. For the sake of clarity, the right panels are only shown for the later times. Interestingly, although the block magnetization is not the optimal measurement the resulted F_C takes values greater than unity. This suggests that such a simple measurement can be used for efficient sensing.

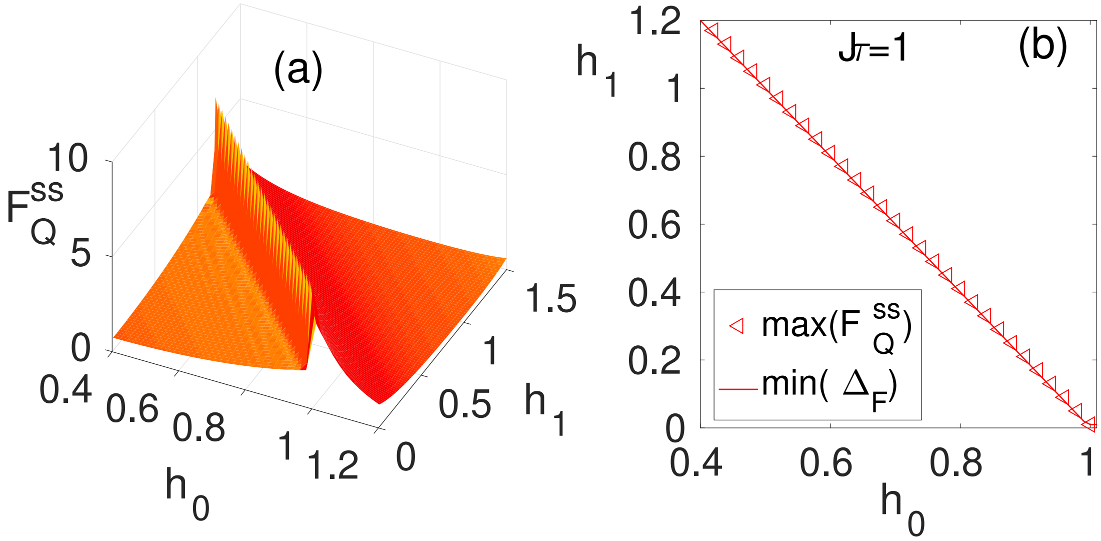

FIG. S2: (a) F_Q^{ss} as a function of h_0 and h_1 for a square pulse magnetic field with $w = \tau/2$ for $L = 4$. (b) For a block size of L=4, maximum of F_Q^{ss} as a function of h_0 and h_1 is depicted by triangles and minimum of Floquet gap is shown with a regular line. Here, we fixed $J\tau$=1 and $N = 2000$.

SENSING SQUARE PULSES

To see the generality of our approach, we also consider square pulsed form of the periodic field, given by the following equation

$$h(t) = \begin{cases} h_1 & \text{if } 0 \leq t \leq w; \\ 0 & \text{if } w \leq t \leq \tau, \end{cases} \tag{S25}$$

where w characterizes the width of the pulse over an interval of τ. The Floquet evolution operator over a time-period is given by

$$U(\tau) = e^{-iH_0(\tau-w)}e^{-i(H_0+h_1\sum_{i=1}^{N}\sigma_i^z)w}. \tag{S26}$$

The local density matrix of the system reaches to a steady state under the AC field of the form given in Eq. (S25). Similar to the case of Dirac-delta kick pulse, the Floquet gap takes its minimum along a straight line on the h_0-h_1 plane and F_Q^{ss} exhibits peaks along the same line. In Fig. S2(a) we plot the steady state quantum Fisher information F_Q^{ss} with respect to h_0 and h_1 for a block of size $L = 4$. The quantum Fisher information shows its peak exactly a straight line. In Fig. S2(b), we plot both max(F_Q^{ss}) and min(Δ_F). The lines of max(F_Q^{ss}) and min(Δ_F) coincides. This shows the generality of the fact that the vanishing Floquet gap results in a higher sensitivity in steady state quantum metrology.

COMPARISON WITH OTHER PROTOCOLS

Now in this section, we outline some of the key points about our protocol addressing its efficacy as compared to other existing protocols for AC-field sensing. We made this comparison with the two main existing schemes, namely spin echo and GHZ-based schemes.

Spin echo utilizes a coherent superposition of spin states and a sequence of external pulses. The typical platform for realizing the spin echo mechanism for AC-field sensing is the nitrogen vacancy centers [S27–S29]. However, the scheme is limited by the maximum time interval needed to accumulate phase and the quality of the coherent superposition of the spin states. To further enhance the precision, one can increase the number of spins. Although, it is crucial that the spins remain non-interacting as any interaction between them acts like decoherence and decreases the precision. In fact, in the case of spin ensembles, the interaction is inevitable and one has to utilize a sophisticated pulse sequence and dynamical decoupling scheme [S30]. Our proposal, however, takes a fundamentally different route as it exploits the interaction between the particles to drive the subsystems into a steady state. This naturally spares us from any

dynamical decoupling scheme. In addition, the enhancement in sensing precision near the vanishing Floquet gap is a resource that helps us to go beyond the standard limit.

Another important feature of the proposed mechanism is its high precision performance despite demanding partial accessibility. Indeed, even with only 1−10 percent accessibility one can still perform high precision sensing. The importance of this becomes even more clear if one compares our protocol with the GHZ-based schemes [S31, S32] in which even losing one particle completely loses its quantumness.

[S1] E. Lieb, T. Schultz, and D. Mattis. Two soluble models of an antiferromagnetic chain. *Ann. Phys.* **16**, 407 (1961).

[S2] E. Barouch and B. M. McCoy. Statistical mechanics of the XY model II. spin-correlation functions. *Phys. Rev. A* **3**, 786 (1971).

[S3] B. Damski and M. M. Rams. Exact results for fidelity susceptibility of the quantum ising model: the interplay between parity, system size, and magnetic field. *J. Phys. A: Math. Theor.* **47**, 025303 (2013).

[S4] T. J. G. Apollaro, G. M. Palma, and J. Marino. Entanglement entropy in a periodically driven quantum ising ring. *Phys. Rev. B* **94**, 134304 (2016).

[S5] I. Peschel and V. Eisler. Reduced density matrices and entanglement entropy in free lattice models. *J. Phys. A: Math. Theor.* **42**, 504003 (2009).

[S6] D. J. Yates and A. Mitra. Entanglement properties of the time-periodic kitaev chain. *Phys. Rev. B* **96**, 115108 (2017).

[S7] A. Carollo, B. Spagnolo, and D. Valenti. Symmetric logarithmic derivative of fermionic gaussian states. *Entropy* **20**, 485 (2018).

[S8] A. Carollo, B. Spagnolo, and D. Valenti. Incompatibility in multi-parameter quantum metrology with fermionic gaussian states. *Proceedings* **12**, 34 (2019).

[S9] M. G. A. Paris. Quantum estimation for quantum Technology. *International Journal of Quantum Information* **07**, 125 (2009).

[S10] C. Bonato, M. S Blok, H. T Dinani, D. W Berry, M. L Markham, D. J Twitchen, and R. Hanson. Optimized quantum sensing with a single electron spin using real-time adaptive measurements. *Nat. Nanotechnol.* **11**, 247 (2016).

[S11] R. S. Said, D. W. Berry, and J Twamley. Nanoscale magnetometry using a single-spin system in diamond. *Phys. Rev. B* **83**, 125410 (2011).

[S12] B. L. Higgins, D. W. Berry, S. D. Bartlett, H. M. Wiseman, and G. J. Pryde. Entanglement-free heisenberg-limited phase estimation. *Nature* **450**, 393 (2007).

[S13] D. W. Berry, B. L. Higgins, S. D. Bartlett, M. W. Mitchell, G. J. Pryde, and H. M. Wiseman. How to perform the most accurate possible phase measurements. *Phys. Rev. A* **80**, 052114 (2009).

[S14] B. L. Higgins, D. W. Berry, S. D. Bartlett, M. W. Mitchell, H. M. Wiseman, and G. J. Pryde. Demonstrating heisenberg-limited unambiguous phase estimation without adaptive measurements. *New J. Phys.* **11**, 073023 (2009).

[S15] G. Vidal, J. I. Latorre, E. Rico, and A. Kitaev. Entanglement in quantum critical phenomena. *Phys. Rev. Lett.* **90**, 227902 (2003).

[S16] A. Russomanno, G E Santoro, and R. Fazio. Entanglement entropy in a periodically driven ising chain. *J. Stat. Mech: Theory Exp.* **2016**, 073101 (2016).

[S17] A. Sen(De), U. Sen, and M. Lewenstein. Nonergodicity of entanglement and its complementary behavior to magnetization in an infinite spin chain. *Phys. Rev. A* **70**, 060304 (2004).

[S18] U. Mishra, D. Rakshit, and R. Prabhu. Survival of time-evolved quantum correlations depending on whether quenching is across a critical point in an XY spin chain. *Phys. Rev. A* **93**, 042322 (2016).

[S19] U. Mishra, R. Prabhu, and D. Rakshit. Quantum correlations in periodically driven spin chains: Revivals and steady-state properties. *J. Magn. Magn. Mater.* **491**, 165546 (2019).

[S20] J. Liu, H. Yuan, X. M. Lu, and X. Wang. Quantum fisher information matrix and multiparameter estimation. *J. Phys. A: Math. Theor.* **53**, 023001 (2019).

[S21] C. Monroe, W. C. Campbell, E. E. Edwards, R. Islam, D. Kafri, S. Korenblit, A. Lee, P. Richerme, C. Senko, and J. Smith, Quantum Simulation of Spin Models with Trapped Ions, *Proceedings of the International School of Physics 'Enrico Fermi,' Course 189*, edited by M. Knoop, I. Marzoli, and G. Morigi, 169-187 (2015).

[S22] J. I. Cirac and P. Zoller, Goals and opportunities in quantum simulation, *Nat. Phys.* **8**, 264 (2012).

[S23] R. Blatt and C. F. Roos, Quantum simulations with trapped ions, *Nat. Phys.* **8**, 277 (2012). *J. Phys. A: Math. Theor.* **53**, 023001 (2019).

[S24] Q. Guo, C. Cheng, Z.-H. Sun, Z. Song, H. Li, Z. Wang, W. Ren, H. Dong, D. Zheng, Y. Zhang, R. Mondaini, H. Fan & H. Wang, Observation of energy-resolved many-body localization, *Nat. Phys.* **17**, 234 (2021).

[S25] M. Gong, G. D. Neto, C. Zha, Y. Wu, H. Rong, Y. Ye, S. Li, Q. Zhu, S. Wang, Y. Zhao, F. Liang, J. Lin, Y. Xu, C.-Z. Peng, H. Deng, A. Bayat, X. Zhu, J.-W. Pan, Experimental characterization of quantum many-body localization transition, arXiv:2012.11521.

[S26] P. Roushan, *et. al.*, Spectroscopic signatures of localization with interacting photons in superconducting qubits, *Science* 358, 1175 (2017).

[S27] R. J. Abraham. Principles of magnetic resonance. c. p. slichter. springer, berlin, 1990, ISBN 3 540 5057 6, 640 pages, DM89.00. *Magn. Reson. Chem.* **28**, 1078 (1990).

[S28] T. Müller, C. Hepp, B. Pingault, E. Neu, S. Gsell, M. Schreck, H. Sternschulte, D. S. Nethl, C. Becher, and M. Atatüre. Optical signatures of silicon-vacancy spins in diamond. *Nat. Commun.* **5**, 3328 (2014).

[S29] J. Hansom, C. H. H. Schulte, C. Le Gall, C. Matthiesen, E. Clarke, M. Hugues, J. M. Taylor, and M. AtatÃijre. Environment-assisted quantum control of a solid-state spin via coherent dark states. *Nat. Phys.* **10**, 725 (2014).

[S30] H. Zhou, J. Choi, S. Choi, R. Land ig, A. M. Douglas, J. Isoya, F. Jelezko, S. Onoda, H. Sumiya, P. Cappellaro, H. S. Knowles, H. Park, and M. D. Lukin. Quantum Metrology with Strongly Interacting Spin Systems. *Phys. Rev. X* **10**, 031003 (2020).

[S31] V. Giovannetti, S. Lloyd, and L. Maccone. Quantum metrology. *Phys. Rev. Lett.* **96**, 160501 (2006).

[S32] H. Cable and J. P. Dowling. Efficient generation of large number-path entanglement using only linear optics and feed-forward. *Phys. Rev. Lett.* **99**, 163604 (2007).
